# (4-Meth­oxy­phen­yl)methanaminium chloride

**DOI:** 10.1107/S1600536811004363

**Published:** 2011-02-16

**Authors:** Riadh Kefi, Zeller Matthias, Cherif Ben Nasr

**Affiliations:** a Laboratoire de Chimie des Matériaux, Faculté des Sciences de Bizerte, 7021 Zarzouna, Tunisia; b Youngstown State University, Department of Chemistry, One University Plaza, Youngstown, Ohio 44555-3663, USA

## Abstract

In the crystal structure of the title salt, C_8_H_12_NO^+^·Cl^−^, the methoxy group of the cation is co-planar with the phenylene moiety with an r.m.s. deviation from the mean plane of only 0.005 Å. The ammonium N atom deviates from this plane by 1.403 (1) Å. In the crystal, the (4-meth­oxy­phen­yl)methan­aminium cations and chloride anions are linked by N—H⋯Cl and C—H⋯O hydrogen bonds, resulting in an open framework architecture with hydrogen-bonded ammonium groups and chloride anions located in layers parallel to (011), separated by more hydrophobic layers with interdigitating anisole groups.

## Related literature

For related compounds, see: Oueslati *et al.* (2005*a* ▶); Ben Gharbia *et al.* (2008 ▶). For hydrogen-bond networks, see: Oueslati *et al.* (2005*b* ▶); Zaouali *et al.* (2009 ▶). For graph-set theory, see: Bernstein *et al.* (1995 ▶). For mesomeric effects in related structures, see: Kefi *et al.* (2006 ▶); El Glaoui *et al.* (2009 ▶). 
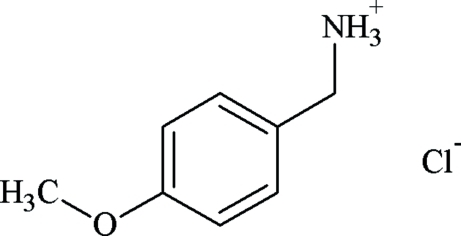


## Experimental

### 

#### Crystal data

C_8_H_12_NO^+^·Cl^−^
*M* *_r_* = 173.64
Monoclinic, 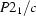

*a* = 11.4234 (11) Å
*b* = 8.9384 (9) Å
*c* = 8.9490 (9) Å
β = 105.904 (1)°
*V* = 878.78 (15) Å^3^
*Z* = 4
Mo *K*α radiation
μ = 0.38 mm^−1^
*T* = 100 K
0.55 × 0.42 × 0.38 mm

#### Data collection

Bruker SMART APEX CCD diffractometer
Absorption correction: multi-scan (*SADABS*; Bruker, 2009 ▶) *T*_min_ = 0.675, *T*_max_ = 0.746
7028 measured reflections
2593 independent reflections
2411 reflections with *I* > 2σ(*I*)
*R*_int_ = 0.015

#### Refinement

*R*[*F* ^2^ > 2σ(*F* ^2^)] = 0.027
*wR*(*F* ^2^) = 0.072
*S* = 1.07
2593 reflections
102 parameters
H-atom parameters constrained
Δρ_max_ = 0.44 e Å^−3^
Δρ_min_ = −0.23 e Å^−3^

### 

Data collection: *APEX2* (Bruker, 2009 ▶); cell refinement: *SAINT* (Bruker, 2009 ▶); data reduction: *SAINT*; program(s) used to solve structure: *SHELXTL* (Sheldrick, 2008 ▶); program(s) used to refine structure: *SHELXTL*; molecular graphics: *SHELXTL*; software used to prepare material for publication: *SHELXTL*.

## Supplementary Material

Crystal structure: contains datablocks global, I. DOI: 10.1107/S1600536811004363/rz2550sup1.cif

Structure factors: contains datablocks I. DOI: 10.1107/S1600536811004363/rz2550Isup2.hkl

Additional supplementary materials:  crystallographic information; 3D view; checkCIF report

## Figures and Tables

**Table 1:** Hydrogen-bond geometry (Å, °)

*D*—H⋯*A*	*D*—H	H⋯*A*	*D*⋯*A*	*D*—H⋯*A*
N1—H1*A*⋯Cl1^i^	0.91	2.24	3.1475 (9)	176
N1—H1*B*⋯Cl1^ii^	0.91	2.25	3.1502 (8)	170
N1—H1*C*⋯Cl1	0.91	2.27	3.1680 (8)	170
C6—H6⋯O1^iii^	0.95	2.58	3.4090 (11)	147

Symmetry codes: (i) 
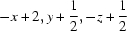
; (ii) 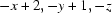; (iii) 
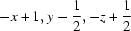
.

**Fig. 1.**
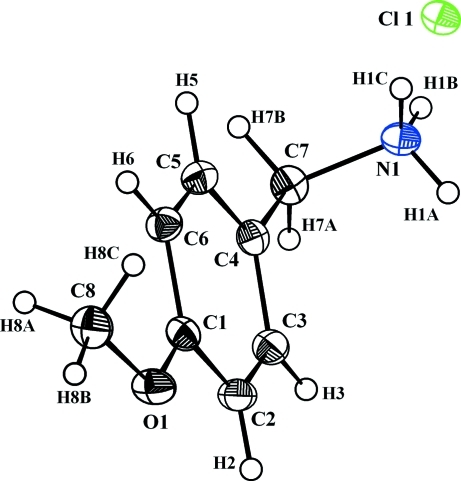
A view of the title compound, showing 60% probability displacement ellipsoids and arbitrary spheres for the H atoms.

**Fig. 2.**
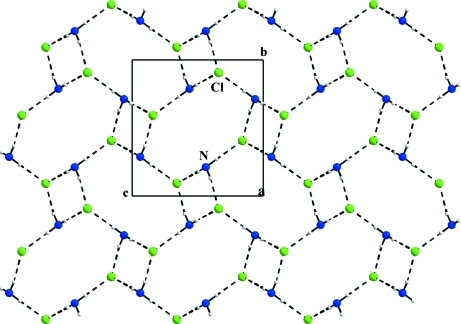
Projection along the a axis of the inorganic layer in the structure of the title compound, showing the N—H···Cl hydrogen bonding interactions (dashed lines). Only the ammonium and chloride sections are shown for clarity.

**Fig. 3.**
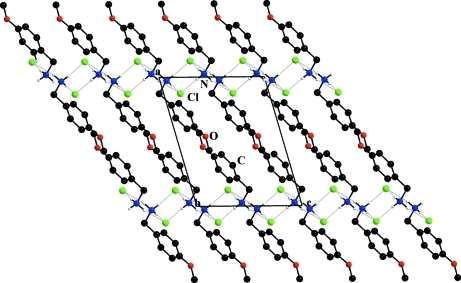
Projection of the structure of the title compound along the b axis. Hydrogen bonds are shown as thin black lines.

## supplementary crystallographic information

### Comment

As a part of our ongoing investigations in molecular salts of amine hydrochloride compounds (Oueslati *et al.*, 2005*a*; Ben Gharbia *et al.*, 2008), we report here the crystal structure of one such compound, (4-methoxyphenyl)methanaminium chloride, C_8_H_12_ClNO (Fig. 1).

The crystal structure consists of a network of the constituent ammonium and chloride ions connected by N—H···Cl hydrogen bonds (Fig. 2), with a chloride anion acting as a threefold acceptor as similarly observed in related compounds (Oueslati *et al.*, 2005*b*). The N···Cl distances vary between 3.1475 (9) and 3.1680 (8) Å, indicating strong interactions between the ammonium and halogenide ions (Zaouali *et al.*, 2009). Multiple hydrogen bonds connect the different entities of the compound to form inorganic layers, built from the chloride anions and the ammonium groups, parallel to the *bc* plane (Fig. 2). Within the layers, various graph-set motifs (Bernstein *et al.*, 1995) are apparent, including R_2_^4^(8) and R_2_^8^(16) motifs. The organic fragments are located between successive inorganic layers (Fig. 3). No π-π stacking interactions between the phenylene rings or C—H···π interactions towards them are observed. A weak intermolecular C—H···O hydrogen interaction involving an aromatic hydrogen atom is present (Table 1). The organic molecule exhibits a regular spatial configuration with usual distances and angles. The distance C1—O1 [1.3637 (11) Å] is slightly shorter than that of C8—O1 [1.4362 (12) Å], which can be attributed to the donor mesomeric effect of the methoxy group. All the geometrical features of the title compound agree with those found in related compounds (e.g. Kefi *et al.*, 2006; El Glaoui *et al.*, 2009).

### Experimental

4-Methoxybenzylamine (2 mmol, 0.274 g) was dissolved in aqueous HCl (10 ml, 1*M*). Colourless crystals suitable for single-crystal X-ray analysis were grown by slow evaporation at room temperature over a period of three weeks (yield 63%).

### Refinement

All H atoms were located in a difference Fourier map, but were repositioned geometrically and refined as riding, with C—H distances of 0.95 (aromatic), 0.99 (methylene) or 0.98 Å (methyl), and N—H distances of 0.91 Å. The torsion angles of the methyl and ammonium H atoms were allowed to refine to best fit the experimental electron density map, and the *U*_iso_(H) values of the these groups were constrained to 1.5 times that of their carrier atom. For the other hydrogen atoms *U*_iso_ was set to 1.2 times *U*_eq_ of the carrier atom.

### Crystal data

**Table:**

C_8_H_12_NO^+^·Cl^−^	*F*(000) = 368
*M**_r_* = 173.64	*D*_x_ = 1.312 Mg m^−^^3^
Monoclinic, *P*2_1_/*c*	Mo *K*α radiation, λ = 0.71073 Å
Hall symbol: -P 2ybc	Cell parameters from 4317 reflections
*a* = 11.4234 (11) Å	θ = 2.3–30.9°
*b* = 8.9384 (9) Å	µ = 0.38 mm^−^^1^
*c* = 8.9490 (9) Å	*T* = 100 K
β = 105.904 (1)°	Block, colourless
*V* = 878.78 (15) Å^3^	0.55 × 0.42 × 0.38 mm
*Z* = 4	

### Data collection

**Table:**

Bruker SMART APEX CCD diffractometer	2593 independent reflections
Radiation source: fine-focus sealed tube	2411 reflections with *I* > 2σ(*I*)
graphite	*R*_int_ = 0.015
ω scans	θ_max_ = 31.0°, θ_min_ = 1.9°
Absorption correction: multi-scan (*SADABS*; Bruker, 2009)	*h* = −15→16
*T*_min_ = 0.675, *T*_max_ = 0.746	*k* = −12→12
7028 measured reflections	*l* = −12→12

### Refinement

**Table:**

Refinement on *F*^2^	Primary atom site location: structure-invariant direct methods
Least-squares matrix: full	Secondary atom site location: difference Fourier map
*R*[*F*^2^ > 2σ(*F*^2^)] = 0.027	Hydrogen site location: inferred from neighbouring sites
*wR*(*F*^2^) = 0.072	H-atom parameters constrained
*S* = 1.07	*w* = 1/[σ^2^(*F*_o_^2^) + (0.0349*P*)^2^ + 0.3154*P*] where *P* = (*F*_o_^2^ + 2*F*_c_^2^)/3
2593 reflections	(Δ/σ)_max_ = 0.001
102 parameters	Δρ_max_ = 0.44 e Å^−^^3^
0 restraints	Δρ_min_ = −0.23 e Å^−^^3^

### Special details

**Table:**

Geometry. All e.s.d.'s (except the e.s.d. in the dihedral angle between two l.s. planes) are estimated using the full covariance matrix. The cell e.s.d.'s are taken into account individually in the estimation of e.s.d.'s in distances, angles and torsion angles; correlations between e.s.d.'s in cell parameters are only used when they are defined by crystal symmetry. An approximate (isotropic) treatment of cell e.s.d.'s is used for estimating e.s.d.'s involving l.s. planes.
Refinement. Refinement of *F*^2^ against ALL reflections. The weighted *R*-factor *wR* and goodness of fit *S* are based on *F*^2^, conventional *R*-factors *R* are based on *F*, with *F* set to zero for negative *F*^2^. The threshold expression of *F*^2^ > σ(*F*^2^) is used only for calculating *R*-factors(gt) *etc*. and is not relevant to the choice of reflections for refinement. *R*-factors based on *F*^2^ are statistically about twice as large as those based on *F*, and *R*- factors based on ALL data will be even larger.

### Fractional atomic coordinates and isotropic or equivalent isotropic displacement parameters (Å^2^)

**Table:**

	*x*	*y*	*z*	*U*_iso_*/*U*_eq_	
Cl1	0.874270 (19)	0.40670 (2)	0.15865 (3)	0.01574 (7)	
O1	0.54020 (6)	1.00932 (8)	0.27742 (9)	0.01962 (15)	
N1	0.97373 (7)	0.71225 (9)	0.06200 (9)	0.01532 (15)	
H1A	1.0144	0.7700	0.1435	0.023*	
H1B	1.0261	0.6811	0.0080	0.023*	
H1C	0.9417	0.6312	0.0982	0.023*	
C2	0.71566 (8)	1.04328 (10)	0.19393 (11)	0.01744 (18)	
H2	0.7270	1.1373	0.2454	0.021*	
C5	0.68479 (8)	0.76756 (10)	0.04614 (11)	0.01565 (17)	
H5	0.6743	0.6728	−0.0039	0.019*	
C1	0.61611 (8)	0.95355 (10)	0.19710 (11)	0.01482 (17)	
C6	0.60029 (8)	0.81501 (10)	0.12293 (11)	0.01576 (17)	
H6	0.5328	0.7536	0.1246	0.019*	
C3	0.79770 (8)	0.99464 (10)	0.11556 (11)	0.01656 (18)	
H3	0.8644	1.0568	0.1124	0.020*	
C7	0.87306 (9)	0.80189 (11)	−0.04294 (11)	0.01650 (17)	
H7A	0.9082	0.8891	−0.0835	0.020*	
H7B	0.8300	0.7394	−0.1325	0.020*	
C4	0.78390 (8)	0.85561 (10)	0.04109 (10)	0.01412 (16)	
C8	0.43276 (9)	0.92405 (12)	0.27309 (13)	0.0218 (2)	
H8A	0.3835	0.9135	0.1652	0.033*	
H8B	0.3852	0.9757	0.3334	0.033*	
H8C	0.4562	0.8247	0.3177	0.033*	

### Atomic displacement parameters (Å^2^)

**Table:**

	*U*^11^	*U*^22^	*U*^33^	*U*^12^	*U*^13^	*U*^23^
Cl1	0.01692 (12)	0.01417 (11)	0.01804 (12)	0.00094 (7)	0.00799 (8)	0.00074 (7)
O1	0.0163 (3)	0.0169 (3)	0.0289 (4)	0.0001 (2)	0.0116 (3)	−0.0034 (3)
N1	0.0178 (4)	0.0137 (3)	0.0164 (3)	0.0002 (3)	0.0080 (3)	−0.0007 (3)
C2	0.0162 (4)	0.0132 (4)	0.0233 (5)	−0.0005 (3)	0.0062 (3)	−0.0019 (3)
C5	0.0175 (4)	0.0148 (4)	0.0148 (4)	−0.0011 (3)	0.0048 (3)	−0.0012 (3)
C1	0.0138 (4)	0.0143 (4)	0.0169 (4)	0.0020 (3)	0.0051 (3)	0.0008 (3)
C6	0.0149 (4)	0.0151 (4)	0.0177 (4)	−0.0019 (3)	0.0051 (3)	−0.0003 (3)
C3	0.0144 (4)	0.0149 (4)	0.0208 (4)	−0.0013 (3)	0.0055 (3)	0.0010 (3)
C7	0.0182 (4)	0.0191 (4)	0.0135 (4)	0.0010 (3)	0.0065 (3)	0.0014 (3)
C4	0.0144 (4)	0.0155 (4)	0.0127 (4)	0.0010 (3)	0.0042 (3)	0.0017 (3)
C8	0.0145 (4)	0.0227 (4)	0.0304 (5)	−0.0001 (3)	0.0096 (4)	−0.0008 (4)

### Geometric parameters (Å, °)

**Table:**

O1—C1	1.3634 (11)	C5—H5	0.9500
O1—C8	1.4362 (12)	C1—C6	1.3932 (13)
N1—C7	1.5015 (12)	C6—H6	0.9500
N1—H1A	0.9100	C3—C4	1.3984 (13)
N1—H1B	0.9100	C3—H3	0.9500
N1—H1C	0.9100	C7—C4	1.5011 (13)
C2—C3	1.3854 (13)	C7—H7A	0.9900
C2—C1	1.3982 (13)	C7—H7B	0.9900
C2—H2	0.9500	C8—H8A	0.9800
C5—C4	1.3897 (13)	C8—H8B	0.9800
C5—C6	1.3954 (13)	C8—H8C	0.9800
			
C1—O1—C8	117.00 (8)	C2—C3—C4	121.10 (8)
C7—N1—H1A	109.5	C2—C3—H3	119.4
C7—N1—H1B	109.5	C4—C3—H3	119.4
H1A—N1—H1B	109.5	C4—C7—N1	111.46 (7)
C7—N1—H1C	109.5	C4—C7—H7A	109.3
H1A—N1—H1C	109.5	N1—C7—H7A	109.3
H1B—N1—H1C	109.5	C4—C7—H7B	109.3
C3—C2—C1	119.80 (8)	N1—C7—H7B	109.3
C3—C2—H2	120.1	H7A—C7—H7B	108.0
C1—C2—H2	120.1	C5—C4—C3	118.31 (8)
C4—C5—C6	121.57 (8)	C5—C4—C7	120.38 (8)
C4—C5—H5	119.2	C3—C4—C7	121.31 (8)
C6—C5—H5	119.2	O1—C8—H8A	109.5
O1—C1—C6	123.91 (8)	O1—C8—H8B	109.5
O1—C1—C2	116.06 (8)	H8A—C8—H8B	109.5
C6—C1—C2	120.02 (8)	O1—C8—H8C	109.5
C1—C6—C5	119.20 (8)	H8A—C8—H8C	109.5
C1—C6—H6	120.4	H8B—C8—H8C	109.5
C5—C6—H6	120.4		
			
C8—O1—C1—C6	−5.30 (13)	C1—C2—C3—C4	−1.01 (14)
C8—O1—C1—C2	175.73 (8)	C6—C5—C4—C3	0.05 (14)
C3—C2—C1—O1	179.65 (8)	C6—C5—C4—C7	−179.76 (8)
C3—C2—C1—C6	0.64 (14)	C2—C3—C4—C5	0.66 (14)
O1—C1—C6—C5	−178.88 (9)	C2—C3—C4—C7	−179.53 (9)
C2—C1—C6—C5	0.05 (14)	N1—C7—C4—C5	−88.82 (10)
C4—C5—C6—C1	−0.40 (14)	N1—C7—C4—C3	91.37 (10)

### Hydrogen-bond geometry (Å, °)

**Table:**

*D*—H···*A*	*D*—H	H···*A*	*D*···*A*	*D*—H···*A*
N1—H1A···Cl1^i^	0.91	2.24	3.1475 (9)	176
N1—H1B···Cl1^ii^	0.91	2.25	3.1502 (8)	170
N1—H1C···Cl1	0.91	2.27	3.1680 (8)	170
C6—H6···O1^iii^	0.95	2.58	3.4090 (11)	147

Symmetry codes: (i) −*x*+2, *y*+1/2, −*z*+1/2; (ii) −*x*+2, −*y*+1, −*z*; (iii) −*x*+1, *y*−1/2, −*z*+1/2.
